# Moving Online: Evidence-Based Programming During COVID

**DOI:** 10.1093/geroni/igab046.081

**Published:** 2021-12-17

**Authors:** Susan Hughes, Andrew DeMott, Gerald Stapleton, Gail Huber

**Affiliations:** 1 University of Illinois at Chicago, Chicago, Illinois, United States; 2 UIC, Chicago, Illinois, United States; 3 Northwestern University, Chicago, Illinois, United States

## Abstract

The COVID pandemic disrupted the way evidence-based health promotion programs (EBPs) are delivered to older adults who were the most at-risk group in terms of mortality and faced unprecedented threats to their independence and physical and mental health. Many organizations stopped in-person EBPS causing older adults to lose access to key social networks and health resources. It is a top public health priority to find new ways to keep older adults connected to their EBPs. Fit & Strong! (F&S!) is a group exercise/health education EBP for older adults with arthritis offered by CBOs in 32 states. CBOs stopped offering F&S! in-person in March 2020. Since the lockdown, we have worked closely with our provider network to develop and pilot a version that is remote/online and live, titled “F&S! @Home”. Instructors deliver F&S! @Home to older adults with minimal technological resources. We created a staging website for both providers/instructors and participants that is used to initiate the classes, enable providers to manage participants, collect data, and share support materials. The pilot began September 2020; since that time 15 classes have been offered to 147 participants. Administration on Community Living falls and arthritis outcomes data are being collected. Preliminary analyses of 45 participants and 8 instructors demonstrate a high rating of the program (mean score of 90.2 out of 100) with no adverse outcomes to date. This presentation will review the process of creating the online adaptation, lessons learned, and will review pre/post outcomes and participant and instructor evaluation feedback.

